
JOURNAL INFORMATION
==============================
NLM Title Abbreviation: Behav Neurol
Journal ID: Behav Neurol
NLM Journal ID: 8914585
Journal ID: BN

Behavioural Neurology

ISSN: 0953-4180
EISSN: 1875-8584
Publisher: Wiley

ARTICLE INFORMATION
==============================
PMCID: PMC5434380
PMID: 21422564
DOI: 10.3233/BEN-2010-0304
Article ID: 253161
Article version: 1
Subjects: Research Article

Long Term Improvements in Activities of Daily Living in Patients with Hemispatial Neglect

Harvey Monika 1 *
Muir Keith 2
Reeves Ian 3
Duncan George 3
Birschel Philip 3
Roberts Margaret 3
Livingstone Katrina 4
Jackson Hazel 5
Hogg Caroline 6
Castle Pauline 6
Learmonth Gemma 7
Rossit Stéphanie 8
1 School of Psychology University of Glasgow Glasgow UK
2 Institute of Neurological Sciences Southern General Hospital Glasgow UK
3 Department of Medicine for the Elderly Southern General Hospital Glasgow UK
4 Stroke Discharge and Rehabilitation Team Southern General Hospital Glasgow UK
5 Stroke Unit Southern General Hospital Glasgow UK
6 Mansion House Unit Glasgow UK
7 Victoria Infirmary Glasgow UK
8 Department of Psychology University of Western Ontario London Canada
*Monika Harvey: m.harvey@psy.gla.ac.uk
Print publication date: 2010
Electronic publication date: 2011 Mar 7
Volume: 23
Issue: 4
First page: 237
Last page: 239
Received 2011 Jan 31; Accepted 2011 Jan 31
Copyright: Copyright © 2010 Hindawi Publishing Corporation and the authors.
Copyright year: 2010
License: This is an open access article distributed under the Creative Commons Attribution License, which permits unrestricted use, distribution, and reproduction in any medium, provided the original work is properly cited.
License URL: https://creativecommons.org/licenses/by/3.0/

==============================
Behavioural Neurology 23 (2010) 237–239
DOI 10.3233/BEN-2010-0304
IOS Press

### Long term improvements in activities of daily living in patients with hemispatial neglect

Monika Harvey[a,*], Keith Muir[b], Ian Reeves[c], George Duncan[c], Philip Birschel[c], Margaret Roberts[c], Katrina Livingstone[d], Hazel Jackson[e], Caroline Hogg[f], Pauline Castle[f], Gemma Learmonth[g] and Stéphanie Rossit[h]

[a]*School of Psychology, University of Glasgow, Glasgow, UK*
[b]*Institute of Neurological Sciences, Southern General Hospital, Glasgow, UK*
[c]*Department of Medicine for the Elderly, Southern General Hospital, Glasgow, UK*
[d]*Stroke Discharge and Rehabilitation Team, Southern General Hospital, Glasgow, UK*
[e]*Stroke Unit, Southern General Hospital, Glasgow, UK*
[f]*Mansion House Unit, Glasgow, UK*
[g]*Victoria Infirmary, Glasgow, UK*
[h]*Department of Psychology, University of Western Ontario, London, Canada*

Stroke is the 3rd commonest cause of death and the most frequent cause of severe adult disability in Scotland (SIGN guidelines, 119, June 2010). Up to 80% of right hemisphere damaged stroke patients experience neglect, an inability to respond to events in the left half of their subjective space. Although the majority of these patients recover spontaneously, the early presence of neglect is the strongest predictor of overall poor recovery from stroke and sub-acute neglect patients have a much-reduced quality of life compared to other stroke patients. Although the last 40 years have seen huge efforts in the field of neglect rehabilitation, what is currently lacking are interventions that demonstrate a generalisation to tasks that assess a patient's ability to function in activities relevant to their and their carer's lives.

In this study we applied visuomotor feedback and control training to a group of patients suffering from hemispatial neglect. The training was based on the established theoretical approach by Milner and Goodale [8]. This model of vision proposes that there are two major parallel visual systems within the brain: the occipito-temporal 'ventral stream' which provides the hardware for analysing the visual input to furnish a rich representation of the scene; and the occipito-parietal 'dorsal stream' (in particular the superior parietal lobe) which mediates the transformation of visual inputs into action. In line with this model, fMRI studies have identified the location of the human homologue of the anterior intraparietal area, a region strongly active during visually guided grasping movements (e.g., Culham et al. [2]). This fits well with a study by Binkofski et al. [1], who demonstrated that lesions in the anterior part of the intraparietal sulcus are associated with impairments in visually-guided grasping. However, lesions resulting in neglect symptoms are typically located more inferiorly in the parietal [7] or even the temporal lobe (Karnath et al. [6]), typically sparing these dorsal stream regions. Secondly, Robertson et al. [9] found that neglect patients showed significantly fewer bisection errors when asked to *grasp* a rod at its midpoint than when asked to *point* to the rod's midpoint. Later studies with groups of neglect patients did indeed find no evidence for deficits in grasping behaviour

*Corresponding author: Monika Harvey, School of Psychology, University of Glasgow, Glasgow G12 8QB, Scotland, UK. Tel.: +44 141 330 6174; Fax: +44 141 330 4606; E-mail: M.Harvey@psy.gla.ac.uk.

ISSN 0953-4180/10/$27.50 © 2010 – IOS Press and the authors. All rights reserved

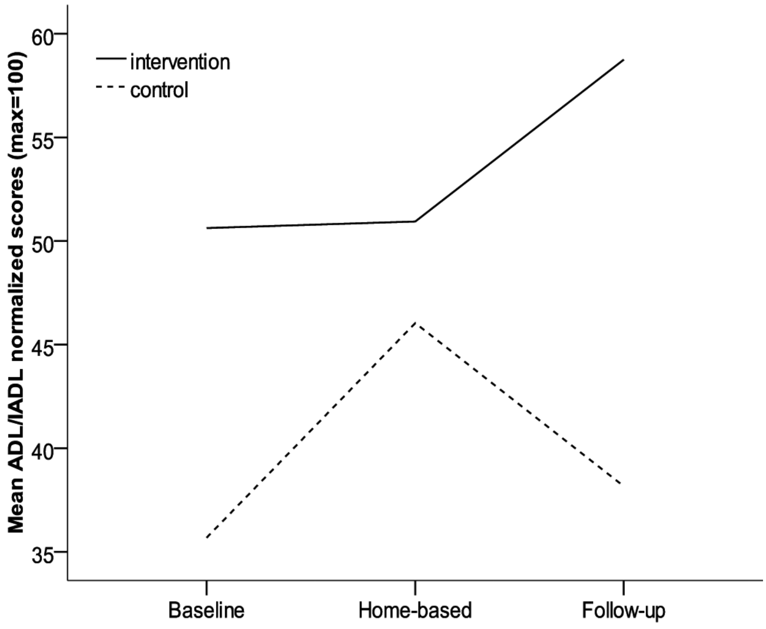

Fig. 1. ADL/IADL scores for intervention and control groups at baseline, after the 2 week home-based training, and at 4 months follow up ($N =$ 15 patients).

in these patients (Harvey et al. [4]), further indicating that the dorsal visual stream may be driving this intact/preserved behaviour. Furthermore, our group (Harvey et al. [5]) found that the simple training of central grasping and lifting responses to left-right oriented rods resulted in significant improvements in chronic neglect patients (average 12 months post stroke).

In the current study, a neglect intervention group was again asked to reach, lift and balance rods at the centre readjusting until satisfied with the judged central grip, whilst the patients in the control group reached and lifted the right-hand side of rods only. The intervention group therefore received proprioceptive, as well as visual feedback, on how well they grasped the centre of the rod; however both groups received a comparable amount of motor experience of reaching and lifting rods. Participants having mastered the exercise for two days, with the experimenter present, embarked on a home-based intervention of 2 weeks, in which they repeated the training independently. Effects of the training were explored with neglect measures (Behavioural Inattention Test; Wilson et al. [10]), but we also assessed the intervention's impact on activities of daily living as measured with the Stroke Impact Scale (Duncan et al. [3]). This outcome assessment was done after the home-based period as well as 4 months post-intervention and compared to the performance before training (baseline).

We found that compared to the control group, the intervention group improved in neglect measures both after the home-based and the 4 months testing period. More importantly, after 4 months the intervention group markedly ameliorated their score in the activities of the daily living domain (ADL/IADL) of the Stroke Impact Scale, whilst the control group reverted to baseline (see Fig. 1).

The ADL/IADL domain of the scale assesses important aspects of the patient's daily routine including eating, dressing, personal hygiene, household tasks, family role and the ability to control their own life. As far as we are aware no controlled intervention has so far been able to generalise recovery to these measures. We postulate that the grasping movements towards objects involved in this form of rehabilitation allow 'leakage' of information of the object via the unaffected dorsal visual stream.

To conclude, visuomotor feedback training reduced neglect symptoms and increased the patients' quality of life. The procedure is easy to apply in any clinical setting (carers, or medical staff could be trained in the space of a couple of hours), non-intrusive (the procedure is behavioural only) and precludes the need of pricey equipment handled by qualified staff. We believe that the current results are most promising and warrant a promotion of this technique in a more general clinical setting.

### Acknowledgments

This work was funded by grants (SFRH/BD/23230/2005; SFRH/BPD/65951/2009) from the Portuguese Foundation for Science and Technology to S Rossit.

### References

[1] F. Binkofski, C. Dohle, S. Posse, K.M. Stephan, H. Hefter, R.J. Seitz and H.J. Freund, Human anterior intraparietal area subserves prehension: a combined lesion and functional MRI activation study, *Neurology* **50** (1998), 1253–1259.

[2] J. Culham, Human brain imaging reveals a parietal area specialized for grasping, in: *Functional Neuroimaging of Visual Cognition: Attention and Performance XX*, N. Kanwisher and J. Duncan, eds, Oxford: Oxford University Press, 2004, pp. 417–438.

[3] P.W. Duncan, R.K. Bode, S.M. Lai and S. Perera, Rasch analysis of a new stroke-specific outcome scale: the Stroke Impact Scale, *Archives of Physical Medicine and Rehabilitation* **84** (2003), 950–963.

[4] M. Harvey, S.R. Jackson, R. Newport, T. Krämer, D.L. Morris and L. Dow, Is grasping impaired in hemispatial neglect? *Behavioural Neurology* **13** (2001), 17–28.

[5] M. Harvey, B. Hood, A. North and I.H. Robertson, The effects of visuomotor feedback training on the recovery of hemispatial neglect symptoms: assessment of a 2-week and follow-up intervention, *Neuropsychologia* **41** (2003), 886–893.

[6] H.-O. Karnath, M.F. Berger, W. Küker and C. Rorden, The anatomy of spatial neglect based on Voxelwise Statistical Analysis: A Study of 140 Patients, *Cerebral Cortex* **14** (2004), 1164–1172.

[7] D.J. Mort, P. Malhotra, S.K. Mannan, C. Rorden, A. Pambakian, C. Kennard and M. Husain, The anatomy of visual neglect, *Brain* **126** (2003), 1986–1997.

[8] A.D. Milner and M.A. Goodale, *The visual brain in action*, Oxford University Press, Oxford, 2006.

[9] I.H. Robertson, D. Nico and B.M. Hood, The intention to act improves unilateral left neglect: two demonstrations, *Neuroreport* **7** (1995), 246–248.

[10] B.A. Wilson, J. Cockburn and P. Halligan, *Behavioural Inattention Test*, Titchfield, Thames Valley Test Company, 1987.
